# Social Determinants of Health Are Associated with Markers of Renal Injury in Adolescents with Type 1 Diabetes

**DOI:** 10.1016/j.jpeds.2018.03.030

**Published:** 2018-07

**Authors:** Laura A.M. Cummings, Antoine Clarke, Etienne Sochett, Denis Daneman, David Z. Cherney, Heather N. Reich, James W. Scholey, David B. Dunger, Farid H. Mahmud

**Affiliations:** 1 Department of Pediatrics, Hospital for Sick Children, University of Toronto, Toronto, Ontario, Canada; 2 Division of Nephrology, University Health Network, University of Toronto, Toronto, Ontario, Canada; 3 Department of Pediatrics, University of Cambridge, Cambridge, United Kingdom

**Keywords:** type 1 diabetes, diabetic kidney disease, social determinants, ACR, Albumin:creatinine ratio, AdDIT, Adolescent Type 1 Diabetes Cardio-Renal Intervention Trial, BMI, Body mass index, eGFR, Estimated glomerular filtration rate, GFR, Glomerular filtration rate, HbA1c, Hemoglobin A1c, IL, Interleukin, MCP, Monocyte chemoattractant protein, MDC, Macrophage-derived chemokine, MIP-1α, Macrophage inflammatory protein-1α, ON-Marg, Ontario Marginalization Index, PDGF, Platelet-derived growth factor, T1D, Type 1 diabetes

## Abstract

**Objective:**

To examine the relationship between the social determinants of health and markers of early renal injury in adolescent patients with type 1 diabetes (T1D).

**Study design:**

Renal outcomes included estimated glomerular filtration rate (eGFR) and albumin–creatinine excretion ratio (ACR). Differences in urinary and serum inflammatory markers also were assessed in relation to social determinants of health. Regression analysis was used to evaluate the association between the Ontario Marginalization Index (ON-Marg) as a measure of the social determinants of health, patient characteristics, ACR, eGFR, and renal filtration status (hyperfiltration vs normofiltration).

**Results:**

Participants with T1D (n = 199) with a mean age of 14.4 ± 1.7 years and diabetes duration of 7.2 ± 3.1 years were studied. Mean eGFR was 122.0 ± 19.4 mL/min/1.73 m^2^. Increasing marginalization was positively associated with eGFR (*P* < .0001) but not with ACR (*P* = .605). Greater marginalization was associated with greater median levels of urinary interleukin (IL)-2, IL-12 (p40), macrophage-derived chemokine, monocyte chemoattractant protein-3, and tumor necrosis factor-β and serum IL-2. ON-Marg was significantly associated with eGFR after we controlled for age, sex, body mass index z score, ethnicity, serum glucose, and hemoglobin A1c in linear regression. A similar association between hyperfiltration and ON-Marg score was observed in multivariable logistic regression.

**Conclusion:**

Increasing marginalization is significantly associated with both eGFR and hyperfiltration in adolescents with T1D and is associated with significant changes in urinary inflammatory biomarkers. These findings highlight a potentially important interaction between social and biological determinants of health in adolescents with T1D.

Diabetic nephropathy is associated with morbidity and mortality in patients with type 1 diabetes (T1D)^1^ and will develop in approximately one-third of patients with T1D. Diabetic nephropathy is a progressive disease that begins with microalbuminuria and later progresses to overt nephropathy, rapid renal decline, and end-stage renal disease.^2^ In this paradigm, microalbuminuria is a key indicator of subsequent risk of diabetic nephropathy. Newer studies have demonstrated that many patients with microalbuminuria do not progress and may even revert to normoalbuminuria.^3^ Glomerular hyperfiltration is similarly considered a risk factor for the development of diabetic nephropathy^4^ and is associated with early markers of renal injury, including albuminuria and increased urinary cytokine/chemokine excretion before the development of microalbuminuria.^5^

Beyond traditional physiological factors associated with diabetic kidney disease, social determinants of health have a powerful influence on outcomes.^6^ The social determinants of health encompass “the conditions in which people are born, grow, work and age, and the systems put in place to deal with illness” and include factors such as education, income, and social status.^7^ These factors are of particular importance during childhood, where exposure to social disadvantage can set young patients on a trajectory that can shape future health outcomes, persisting long into adulthood.^8^, ^9^, ^10^, ^11^ Most research in the field of social medicine has focused on early childhood exposures and experiences, yet adolescence is an equally critical developmental period that is highly sensitive to the effects of social determinants of health.^9^ Despite the growing repertoire of technological innovations in diabetes care, adolescents continue to exhibit suboptimal glycemic control compared with adults.^12^ Adolescence is also a critical period for determining the lifetime risk of complications in T1D due to hormonal and metabolic changes and because the first signs of microvascular disease appear during this time.^13^, ^14^, ^15^, ^16^

Many behavioral and biological outcomes in children with T1D track along a social gradient,^17^ including poorer psychosocial functioning,^18^ worsened glycemic control,^19^, ^20^ greater risk of acute complications including diabetic ketoacidosis and acute care use,^21^ increased prevalence of modifiable cardiovascular risk factors, and early signs of cardiovascular dysfunction.^19^ The goal of our study was to explore the relationships between social determinants of health and signs of early renal risk in pediatric patients with T1D. We also sought to examine potential differences in urinary and serum inflammatory markers in relation to social determinants of health as a potential biological mediator linking marginalization with early renal changes.

## Methods

This study evaluated participant data from an existing cohort of adolescents with T1D from the observational arm of the Canadian Adolescent Diabetes Cardiorenal Intervention Trial (AdDIT),^22^, ^23^ linked to population-level census data from Ontario, Canada. Patient-level data were obtained from participants with T1D receiving care at the Hospital for Sick Children and affiliated regional diabetes care centers in Toronto, Ontario, who were enrolled in the observational arm of the AdDIT clinical trial. All data used for this study were obtained at the participants' baseline study visit. The Ontario Marginalization Index (ON-Marg) was used as an area-level measure of the social determinants of health. Ethics approval for the AdDIT study was granted through the Hospital for Sick Children institutional research ethics board.

Detailed descriptions of the AdDIT study population and methods have been published previously.^22^, ^23^ This population has also been shown to be representative of the greater Toronto population in terms of the social determinants of health.^24^

### ON-Marg Measures

The ON-Marg is a census-based index developed to assess levels of marginalization across residential areas in Ontario, Canada. This measure has been validated across time and geographic areas and has proven to be a useful tool for the study of health disparities, being associated with numerous health outcomes.^25^ The ON-Marg represents the average of 4 social determinants of health dimensions measured in quintiles, each representing 20% of the reference population (Q1: least marginalized; Q5: most marginalized).^26^ The 4 dimensions include residential instability (ie, housing status, home ownership, etc), material deprivation (ie, education, unemployment, income, etc), ethnic concentration (ie, recent immigrants, visible minorities, etc), and dependency (ie, participation in labor force). Details of ON-Marg indicators, dimensions, and use are available from the ON-Marg User Guide.^26^

### Primary and Secondary Outcomes

The primary outcomes of this study were markers of early renal injury, assessed by participants' baseline estimated glomerular filtration rate (eGFR) and albumin:creatinine ratio (ACR). eGFR was calculated via a combined cystatin-C and creatinine-based equation (eGFR_Zappitelli_), which has demonstrated accuracy in estimating glomerular filtration rate (GFR) in various pediatric populations, including patients with and without renal disease.^27^, ^28^, ^29^ ACR was determined based on 2 sets of 3 early morning urine samples.

The secondary outcome of this study was baseline inflammation, assessed by serum and urinary levels of 15 inflammatory markers. These markers were selected a priori from a list of 47 analytes previously measured as part of the AdDIT study and included eotaxin, fibroblast growth factor-2, interferon-α, interleukin (IL)-2, IL-6, and IL-12 (p40/p70), macrophage-derived chemokine (MDC), monocyte chemoattractant protein (MCP)-1, MCP-3, macrophage inflammatory protein-1α (MIP-1α), tumor necrosis factor (TNF)-α, TNF-β, Scd4-ligand (sCD40L), platelet-derived growth factor (PDGF)-AA, PDGF-BB, and regulated on activation, normal T-cell expressed and secreted (RANTES). Selection was based on analytes previously associated with hyperfiltration in similar populations^5^, ^30^, ^31^ or with socioeconomic status.^32^ Details regarding AdDIT study baseline biochemical and clinical assessments have been described previously.^22^, ^23^, ^31^ Urinary cytokine values were adjusted for urine creatinine to account for differences in concentration. Biochemical data outside assay limits of detection were not included for statistical analysis.

### Other Variables of Interest

Other variables included in our analysis as potential modifiers were sex, age at baseline, duration of T1D, ethnicity (white vs nonwhite), treatment regime (pump vs injection therapy), glycemic control (hemoglobin A1c [HbA1c]), height, weight, waist circumference, body mass index (BMI) z scores, lipids (high-density lipoprotein, low-density lipoprotein, triglycerides, cholesterol), blood pressure, and smoking status. All variables were recorded from baseline clinic visits.^22^

Although more detailed data regarding patient ethnicity were available (white, black, Chinese, South Asian, and other), ethnicity was dichotomized as white vs nonwhite, given the relatively small sample size of the other ethnic groups in this cohort. The low statistical power associated with the analysis of individual ethnic groups limited the opportunity to extrapolate meaningful conclusions from participant data, such that dichotomization of the ethnicity variable was used for analytical purposes.

### Statistical Analyses

Statistical analysis was carried out with R Statistics v.3.1.2 (R Foundation for Statistical Computing, Vienna, Austria; www.R-project.org). Continuous characteristics were summarized by the use of summary statistics (mean ± SD); categorical variables were summarized with frequencies and percentages. The Pearson correlation was used to assess correlations between ON-Marg quintile scores and eGFR, ACR, and urinary/serum markers. For correlations with a consistent directionality across ON-Marg dimensions, we further assessed correlations between outcomes and ON-Marg composite score, as per ON-Marg user guidelines.^26^ Given the diverse ethnic concentration in Toronto, correlation with ON-Marg summary score also was assessed for variables with consistent directional correlations with all dimensions except for ethnic concentration. All tests were conducted with a significance level of *P* < .05. *P* values for correlation tests between cytokines and ON-Marg were adjusted with the Benjamini–Hochberg correction for multiple comparisons.

Linear regression analysis with backward stepwise selection was used to assess the association between ON-Marg summary score and eGFR, controlling for age, sex, and BMI z score; simple linear regression was used to identify other clinical variables for addition to the model, using a liberal *P* value cutoff of *P* < .20 as a criterion for inclusion. All significant variables identified through this process were added to the model and subsequently eliminated as per the backward stepwise approach, using a *P* value cutoff of *P* < .05. All variables in the final model were assessed for interaction.

To further describe the potential influence of clinical variables and social determinants of health on clinically relevant differences in eGFR, patients were stratified based on filtration status (normofiltration vs hyperfiltration). Hyperfiltration was defined by eGFR values greater or equal to eGFR_Zappitelli_ ≥126.8 mL/min/1.73 m^2^, and normofiltration encompassed all eGFR values below this threshold (eGFR_Zappitelli_ <126.8 mL/min/1.73 m^2^).^33^ This cutoff value was derived from previous work by Fadrowski et al,^29^ which evaluated multiple eGFR measures, including Zappitelli, with regard to National Health and Nutrition Examination Survey data from adolescents between 12 and 17 years of age. The eGFR used herein to define hyperfiltration corresponds to the 95th percentile for eGFR based on this representative dataset. Clinical characteristics and social determinants of health variables were compared between hyperfiltering vs normofiltering patients via the use of 2-sample *t* tests or nonparametric tests; Welch *t* tests or Fisher exact tests were used where applicable. Wilcoxon or χ^2^ tests were used alternatively when parametric tests were not appropriate. We also compared levels of serum and inflammatory markers between normofiltering vs hyperfiltering patients using 2-sample Wilcoxon tests to further explore potential biological pathways that may have influenced observed relationships between social factors and renal function. All tests were conducted at a significance level of *P* < .05.

Logistic regression using the same backward stepwise approach as for our linear model was used to develop a model linking clinical factors and social determinants of health to hyperfiltration. Our final logistic regression model was confirmed via a best subset selection.

## Results

Participants (n = 199) were included in the observational arm of the Canadian AdDIT cohort. Clinical and demographic data were available for all (Table I). The average age was 14.4 ± 1.66 years, with an average diabetes duration of 7.24 ± 3.14 years. Average HbA1c was 8.49 ± 1.24%. Mean eGFR_Zappitelli_ and ACR were 122.0 ± 19.4 mL/min/1.73 m^2^ and 1.10 ± 2.17 mg/mmol, respectively.

**Table I:** Patient characteristics and urinary markers of inflammation from the AdDIT observational cohort, with comparison of normofiltring vs hyperfiltering patients

Characteristics	Total (n = 199)	eGFR_Zappitelli_ >126.8 mL/min/1.73 m^2^		*P* value*
		NF (n = 127)	HF (n = 72)	
Age, y	14.4 (1.66)	14.6 (1.6)	14.1 (1.7)	NS (.054)
T1D duration, y	7.24 (3.14)	7.4 (3.2)	7.0 (3.0)	NS
BMI z score	0.65 (0.90)	0.73 (0.87)	0.51 (0.96)	NS
WC, cm	74.6 (11.8)	76.5 (10.3)	71.3 (13.5)	.006
HbA1c, %	8.49 (1.24)	8.3 (1.2)	8.8 (1.3)	.0049
ACR, mg/mmol	1.10 (2.17)	0.5	0.7	.021
eGFR_Zappitelli_, mL/min/1.73 m^2^	122.0 (19.4)	—	—	—
HDL, mmol/L	1.64 (0.36)	1.61 (0.33)	1.68 (0.38)	NS
LDL, mmol/L	2.30 (0.71)	2.32 (0.75)	2.27 (0.64)	NS
Triglycerides, mmol/L	0.84 (0.37)	0.85 (0.35)	0.82 (0.39)	NS
SBP, mm Hg	115.0 (10.7)	116.2 (11.1)	112.8 (9.6)	.02
DBP, mm Hg	67.5 (7.24)	67.2 (7.1)	67.9 (7.5)	NS
ON-Marg	2.86 (0.75)	2.7 (0.7)	3.1 (0.8)	.0009
Female sex	101 (50.8)	57 (44.9)	44 (61.1)	.0278
Pump therapy	121 (60.8)	83 (65.4)	38 (52.8)	NS (.08)
Nonwhite ethnicity	83 (41.7)	43 (33.9)	40 (55.6)	.0029
Urinary markers				
Eotaxin	—	0.79 (0.60-1.04)	0.94 (0.63-1.41)	.02
FGF-2	—	1.77 (1.26-2.87)	2.40 (1.58-3.96)	.042
IFN-α	—	0.62 (0.38-0.98)	1.02 (0.64-1.61)	.0004
IL-2	—	0.061 (0.038-0.093)	0.093 (0.045-0.137)	.025
IL-6	—	0.084 (0.046-0.131)	0.123 (0.056-0.317)	.0135
IL-12 (p40)	—	0.494 (0.328-0.819)	0.586 (0.404-1.025)	.037
IL-12 (p70)	—	0.047 (0.026-0.088)	0.065 (0.043-0.119)	NS
MDC	—	0.708 (0.492-1.08)	0.952 (0.699-0.1.473)	.0051
MCP-1	—	33.74 (25.52-46.48)	43.88 (29.22-69.30)	.0071
MCP-3	—	0.826 (0.614-1.207)	1.149 (0.704-1.868)	.0151
MIP-1α	—	0.502 (0.291-0.821)	0.625 (0.321-1.099)	NS (.084)
TNF-α	—	0.020 (0.012-0.029)	0.020 (0.011-0.037)	NS
TNF-β	—	0.11 (0.07-0.17)	0.14 (0.08-0.21)	NS (.085)
sCD40L	—	0.131 (0.088-0.228)	0.181 (0.124-0.335)	.0149
PDGF-AA	—	8.45 (6.11-11.49)	10.72 (7.06-15.32)	.0196
PDGF-BB	—	1.54 (1.16-2.18)	2.11 (1.22-3.90)	.03
RANTES	—	1.21 (0.92-1.76)	1.69 (1.00-3.18)	.0026

*DBP*, diastolic blood pressure; *FGF-2*, fibroblast growth factor-2; *HDL*, high-density lipoprotein; *HF*, hyperfiltration; *IFN-α*, interferon-α; *LDL*, low-density lipoprotein; *NF*, normofiltration; *NS*, not significant; *RANTES*, regulated on activation, normal T-cell expressed and secreted; *SBP*, systolic blood pressure; *WC*, waist circumference.

Values are mean (SD), frequency (%), or median (IQR).

* *P* < .05.

### Correlations between ON-Marg Scores and Renal Measures

eGFR_Zappitelli_ was significantly correlated with multiple social determinants of health dimensions and ON-Marg summary score (R = 0.323; *P* < .0001). ACR was significantly inversely correlated with ethnic concentration but was not correlated with ON-Marg summary score (Table II).

**Table II:** Pearson correlations between ON-Marg scores and renal function (n **=** 199)

ON-Marg dimensions	ACR, mg/mmol		eGFR_Zappitelli_, mL/min/1.73 m^2^	
	R	*P*	R	*P*
Dependency	0.075	.29	0.092	.195
Material deprivation	0.0063	.93	0.229	.00114
Residential instability	0.035	.62	0.165	.0202
Ethnic concentration	−0.148	.037	0.190	.0073
Summary score	0.037	.605	0.323	<.0001

### Ethnicity

Significant differences were observed between the white and nonwhite ethnic groups in relation to HbA1c (*P* = .027), ON-Marg (*P* < .01), and Zappitelli eGFR (*P* < .001) but not ACR (*P* = .06). In comparison, no individual ethnic groups within the nonwhite group (ie, black, Chinese, South Asian, and other) were found to be significantly different from one another with respects to ON-Marg and Zappitelli eGFR.

### Linear Regression

Linear regression was undertaken to further explore the relationship between eGFR and ON-Marg summary score while we controlled for other variables. The final model included age, sex, BMI (z score), ethnicity, glucose and HbA1c, and ON-Marg. Although HbA1c was not significantly associated with eGFR when step-wise regression was used, it was retained in the final model as a potential confounder, given its association with socioeconomic status.^17^, ^19^, ^20^ There was a significant positive association between marginalization and eGFR that persisted after we controlled for these other variables (β = 6.1, *P* = .0003) (Table III).

**Table III:** Step-wise backwards multivariable linear and logistic regression modeling to assess the relationship between eGFR, ON-Marg summary score, and other variables of interest

Variables	Linear regression Zappitelli eGFR (mL/min/1.73 m^2^)				Logistic regression NF vs HF			
	β	SE	*P*	Adj R^2^	β	SE	*P*	Adj R^2^
Intercept	114.3	13.6	<.0001	—	−3.09	1.88	.1001	—
Age at baseline	−2.4	0.7	.00083	0.04	−0.20	0.10	.047	0.016
Sex [female]	11.0	2.4	<.0001	0.074	0.90	0.39	.0082	0.03
BMI z score	−4.0	1.4	.0034	0.03	—	—	—	—
Ethnicity [nonwhite]	7.3	2.5	.0033	0.03	0.67	0.34	.047	0.015
Glucose, mmol/L	0.8	0.3	.0027	0.03	0.09	0.04	.025	0.02
HbA1c, %	1.3	1.0	.174	0.003	0.25	0.13	.062	0.014
ON-Marg	6.1	1.6	.00029	0.04	0.54	0.22	.017	0.022
Adjusted R^2^	0.278				0.1406			

Continuous eGFR_Zappitelli_ was used for the linear model whereas the logistic model compared subjects with normofiltration (n = 127; eGFR_Zappitelli_ < 126.8 mL/min/1.73 m^2^) with those with hyperfiltration (n = 72; eGFR_Zappitelli_ ≥ 126.8 mL/min/1.73 m^2^).

### Comparison of Hyperfilterers and Normofilterers

Of 199 patients, 72 were classified as having hyperfiltration using our established cutoff of eGFR_Zappitelli_ > 126.8 mL/min/173 m^2^ (Table I). Patients with hyperfiltration were more likely to be of male sex and nonwhite ethnicity and had significantly greater ACR, ON-Marg summary score, and material deprivation vs patients with normofiltration (*P* < .05) (Table I). Levels of all urinary markers were significantly greater in patients with hyperfiltration relative to patients with normofiltration, with the exception of MIP-1α, TNF-α, and TNF-β (Table I). Serum levels of sCD40L (*P* = .0048) and PDGF-BB (*P* = .0053) were significantly greater in patients with hyperfiltration; other changes in serum markers between groups were not significant (Table IV shows data for all markers; available at www.jpeds.com).

### Associations between ON-Marg and Inflammatory Markers

A minimum of 167 samples were available for all urinary analytes (Table IV). Urinary concentrations of eotaxin, interferon-α, IL-2, IL-12 (p70), MDC, MIP-1α, TNF-β, PDGF-AA, and PGDF-BB levels were positively and consistently associated with increasing marginalization in all 4 ON-Marg dimensions (data not shown). After we applied the Benjamin–Hochberg correction for multiple comparisons, ON-Marg remained significantly correlated with urinary IL-2 (r = 0.197, *P* = .034), IL-12 (p40) (r = 0.199, *P* = .034), MDC (r = 0.195, *P* = .034), MCP-3 (r = 0.200, *P* = .034), and TNF-β (r = 0.183, *P* = .042).

A minimum of 120 samples was available for all serum analytes (Table IV). Serum IL-2, sCD40L, and PDGF-BB were correlated with increased measures of marginalization across all 4 ON-Marg dimensions (data not shown). Only IL-2 remained significantly correlated with ON-Marg after correction for multiple comparisons (r = 0.296, *P* = .003). Remaining serum and urinary markers were inconsistently related with ON-Marg dimensions and therefore were not assessed for correlations with ON-Marg summary scores.

### Logistic Regression

Consistent with our linear regression model, ON-Marg summary score was significantly associated with hyperfiltration after we controlled for other patient factors including age, sex, ethnicity, HbA1c, and glucose in logistic regression (β = 0.54, *P* = .017) (Table III).

### Sensitivity Analysis

A sensitivity analysis was conducted to evaluate the relationships between filtration rate and ON-Marg scores using the Larsson equation, another cystatin C–derived method of GFR estimation validated for use in pediatric populations.^34^ A highly significant association also was observed using this alternative measure (*P* < .0001; Table V; available at www.jpeds.com). Larsson and Zappitelli eGFR measures were comparable in our sample and were highly correlated (R = 0.86; *P* < .0001).

## Discussion

Renal hyperfiltration has been associated with changes in cardiovascular function, including endothelial dysfunction, arterial stiffness and calcification, and reduced exercise capacity.^35^, ^36^, ^37^, ^38^, ^39^ In the pathogenesis of microvascular and macrovascular complications in T1D, inflammatory pathways are implicated as a common final mechanism of end-organ injury.^40^ We observed that increasing social determinants of health by ON-Marg scores were positively associated with urinary concentrations of IL-2, IL-12 (p40), MDC, MCP-3, and TNF-β and with serum IL-2. The inflammatory changes which we observed in association with ON-Marg scores are consistent with the inflammatory profile of renal hyperfiltration in T1D that has been described previously.^30^, ^31^ Moreover, we observed a significant increase in almost all urinary markers in patients with hyperfiltration. Although differences in serum markers based after eGFR stratification were less robust, serum sCD40L was increased in patients with hyperfiltration, which has been identified as a marker of microvascular complication risk.^41^ We did not observe broader associations between social adversity with increased circulating markers of chronic inflammation such as IL-6, TNF-α, and MCP-1 seen in adult studies,^32^, ^40^ nor were we able to assess the temporality of these associations in the current cross-sectional evaluation. Nonetheless, further longitudinal evaluation remains a valuable avenue for future research to assess whether inflammatory changes precede clinical hyperfiltration or microalbuminuria.

Socioeconomic deprivation is a potential risk factor for the development of nondiabetic renal disease.^42^ This study suggests that differences in social determinants of health are associated with eGFR and hyperfiltration in adolescents with T1D. Given the recognized role of hyperfiltration as an early indicator of nephropathy, our findings highlight the potential influence of social factors on renal risk in pediatric diabetes. Previous findings from the AdDIT patient cohort also have linked poorer social determinants of health with cardiovascular risk factors and early vascular dysfunction.^24^ The associations between social determinants of health and renal injury reported in the current study are aligned with these findings and add to our understanding of the impact of social circumstance as a “third arm” of T1D complication risk in addition to modifiable and non-modifiable physiological risk factors.^43^, ^44^

Our analyses also identified associations between eGFR, filtration status, and variables other than marginalization. We observed an inverse association between patient age and eGFR, whereas female sex was associated with both eGFR and hyperfiltration (Table II and Table III). Although male sex has been associated previously with an increased risk of renal complications in adults with T1D, studies in children suggest that the risk of hyperfiltration, persistent microalbuminuria, and end-stage renal disease is greater in young females.^13^, ^30^, ^45^ There was a positive association between serum glucose and eGFR; the association between ON-Marg summary score and eGFR remained significant when glucose was included as a covariable in regression modeling. Similarly, although glucose levels were significantly greater in patients with hyperfiltration, controlling for glucose in logistic regression did not negate the association between ON-Marg summary scores and hyperfiltration. HbA1c was not significantly different between normofiltering vs hyperfiltering patients, and associations between eGFR and hyperfiltration with ON-Marg persisted when HbA1c was added in both models. Although poor glycemic control is a recognized risk factor for diabetic renal disease, differences in glycemic control and/or transient hyperglycemia do not fully account for the observed relationship between eGFR, filtration status, and social marginalization in this study.^39^

Minority youth are disproportionately at risk for diabetes-related complications and mortality.^14^, ^17^ Nonwhite ethnicity was positively associated with both eGFR and hyperfiltration, which persisted in multivariate regression controlling for glucose/HbA1c and BMI z scores. Although minority youth are at a greater risk of obesity and suboptimal glycemic control, socioeconomic disadvantage, psychosocial factors, and genetic/biological differences are important mediators of ethnic differences in T1D outcomes.^14^, ^17^, ^46^, ^47^ An important limitation of this study is that participants were dichotomized broadly into white vs nonwhite ethnicities; this broad classification was required to improve the statistical power of our analyses and to appreciate meaningful differences between groups. We recognize that evaluations of patient cohorts with detailed descriptions of ethnicity are important to understand potential racial and ethnic disparities in early renal injury. More detailed classifications of ethnicity based on Canadian Census definitions^25^ are available in Table VI (available at www.jpeds.com).

We observed an inverse association between BMI z scores and eGFR_Zappitelli_ in both univariable vs multivariable analysis, although it only reached significance in multivariable modelling. BMI was not associated with HbA1c; removal of HbA1c from multivariable modeling did not impact the relationship between BMI and eGFR. Although several authors have noted a similar inverse relationship between BMI scores and eGFR_Zappitelli_ in healthy children and adolescents,^29^, ^48^ more data are needed to assess how BMI affects the distribution of equations for GFR estimation in pediatric T1D cohorts. We also did not observe a significant association between hyperfiltration and BMI, consistent with findings in adult populations.^30^ This lack of association merits further study in light of the established role of obesity as a risk factor for diabetic complications, including cardiovascular and renal disease.

This study has limitations. We acknowledge that using ON-Marg as a proxy for individual socioeconomic status may fail to capture individual-level heterogeneity; however, the ON-Marg provides a robust and valid assessment of social determinants for our population.^49^, ^50^ We used one among a number of equation-based eGFR measures, but the combined cystatin C and creatinine-based Zappitelli equation was purposefully selected for this study; it is known to perform well in pediatric populations and patients with T1D.^27^, ^29^, ^48^, ^51^, ^52^, ^53^ The positive associations between eGFR and ON-Marg scores described here were also found using the Larsson measure of eGFR,^34^ which provides a valid estimation of GFR in various pediatric patients with and without renal disease.^27^, ^28^ This analysis demonstrated that the significant association between ON-Marg summary score and renal filtration is robust using various validated eGFR measures (Table V).

In summary, we report that marginalization, as a measure of social determinants of health, is associated with both eGFR and hyperfiltration in adolescents with T1D and with significant changes in urinary inflammatory profile. The aim of this study was further to describe the influence of social factors on relevant diabetes risk markers. By examining social determinants of health through this lens, this study supports the recognition of social circumstance as a risk factor for the development of later complications and adverse outcomes in chronic disease.^44^ Future research should focus on exploring how neighborhood-level variables influence patient-level outcomes, taking into account both contextual factors and measures of individual social determinants of health.^50^

## Figures and Tables

**Table IV:** Comparison of urinary/serum markers of inflammation based on filtration status (NF vs HF)

Inflammatory markers, mmol/L	eGFR_Zappitelli_ >126.8 mL/min/1.73 m^2^				
	NF		HF		*P*
	n	Median (IQR)	n	Median (IQR)	
Urinary					
Eotaxin	115	0.79 (0.60-1.04)	68	0.94 (0.63-1.41)	.02
FGF-2	111	1.77 (1.26-2.87)	66	2.40 (1.58-3.96)	.042
IFN-α	115	0.62 (0.38-0.98)	68	1.02 (0.64-1.61)	.0004
IL-2	114	0.061 (0.038-0.093)	64	0.093 (0.045-0.137)	.025
IL-6	108	0.084 (0.046-0.131)	64	0.123 (0.056-0.317)	.0135
IL-12 (p40)	115	0.494 (0.328-0.819)	68	0.586 (0.404-1.025)	.037
IL-12 (p70)	105	0.047 (0.026-0.088)	62	0.065 (0.043-0.119)	.16
MDC	114	0.708 (0.492-1.08)	68	0.952 (0.699-0.1.473)	.0051
MCP-1	115	33.74 (25.52-46.48)	68	43.88 (29.22-69.30)	.0071
MCP-3	120	0.826 (0.614-1.207)	63	1.149 (0.704-1.868)	.0151
MIP-1α	111	0.502 (0.291-0.821)	66	0.625 (0.321-1.099)	.084
TNF-α	113	0.020 (0.012-0.029)	67	0.020 (0.011-0.037)	.597
TNF-β	115	0.11 (0.07-0.17)	68	0.14 (0.08-0.21)	.085
sCD40L	110	0.131 (0.088-0.228)	64	0.181 (0.124-0.335)	.0149
PDGF-AA	115	8.45 (6.11-11.49)	68	10.72 (7.06-15.32)	.0196
PDGF-BB	114	1.54 (1.16-2.18)	68	2.11 (1.22-3.90)	.03
RANTES	115	1.21 (0.92-1.76)	68	1.69 (1.00-3.18)	.0026
Serum					
Eotaxin	118	81.3 (57.0-108.2)	70	79.9 (52.7-101.4)	.68
FGF-2	113	35.8 (24.9-66.5)	69	39.9 (23.3-62.2)	.671
IFN-α	102	35.0 (15.0-65.8)	62	28.5 (13.1-65.7)	.36
IL-2	100	5.39 (1.66-11.8)	54	3.73 (1.46-10.77)	.369
IL-6	104	3.47 (1.38-6.69)	61	3.20 (1.50-9.01)	.545
IL-12 (p40)	95	61.0 (18.8-161.5)	49	42.7 (19.2-132.8)	.578
IL-12 (p70)	117	4.01 (2.42-8.29)	70	4.47 (2.80-10.04)	.15
MDC	118	1195.9 (923.8-1513.2)	70	1313.0 (1010.2-1560.5)	.147
MCP-1	118	349.3 (272.5-447.2)	70	327.0 (231.6-501.2)	.597
MCP-3	76	35.9 (19.9-72.9)	44	38.6 (19.9-59.9)	.959
MIP-1α	99	11.4 (6.6-17.8)	60	11.4 (6.7-19.6)	.772
TNF-α	118	9.85 (7.00-12.52)	70	9.90 (7.79-13.01)	.395
TNF-β	81	25.9 (8.8-54.8)	46	31.9 (11.8-71.3)	.353
sCD40L	118	5606.2 (2492.1-15787.7)	70	12645.9 (4252.7-17826.7)	.0048
PDGF-AA	118	1698.8 (1081.0-1967.8)	70	1866.6 (1209.3-2005.3)	.269
PDGF-BB	118	8802.2 (7357.2-10301.8)	70	9968.0 (8528.5-10566.4)	.0053
RANTES	116	1776.1 (1180.0-2616.7)	68	1966.9 (1391.7-3012.4)	.142

*RANTES*, regulated on activation, normal T cell expressed and secreted.

**Table V:** Sensitivity analysis comparing the association between eGFR and ON-Marg scores using the Larsson equation vs Zappitelli equation for estimating GFR

ON-Marg dimensions	ACR, mg/mmol		eGFR Zappitelli, mL/min/1.73 m^2^		eGFR Larsson, mL/min/1.73 m^2^	
	R	*P*	R	*P*	R	*P*
Dependency	0.075	.29	0.092	.195	0.0416	.559
Material deprivation	0.0063	.93	0.229	.00114	0.214	.0025
Residential instability	0.035	.62	0.165	.0202	0.173	.147
Ethnic concentration	−0.148	.037	0.190	.0073	0.122	.0864
Summary score	0.037	.605	0.323	<.0001	0.280	<.0001

**Table VI:** Detailed breakdown of participant ethnicity (based on Canadian Census classification)

Ethnic groups	n	%
White	116	58.3
Black, Afro-Caribbean	15	7.5
Chinese	14	7.0
South Asian	18	9.1
Other and mixed	36	18.1
Total	199	100
